# Vanadium (V) Adsorption from Aqueous Solutions Using Xerogel on the Basis of Silica and Iron Oxide Matrix

**DOI:** 10.3390/ma15248970

**Published:** 2022-12-15

**Authors:** Florin Matusoiu, Adina Negrea, Mihaela Ciopec, Narcis Duteanu, Petru Negrea, Paula Ianasi, Cătălin Ianasi

**Affiliations:** 1 Faculty of Industrial Chemistry and Environmental Engineering, Polytechnic University of Timişoara, Victoriei Square, no. 2, 300006 Timisoara, Romania; 2 National Institute for Research and Development in Electrochemistry and Condensed Matter, 144th Dr. A.P. Podeanu Street, 300569 Timisoara, Romania; 3 “Coriolan Drăgulescu” Institute of Chemistry, Bv. Mihai Viteazul, No. 24, 300223 Timisoara, Romania

**Keywords:** vanadium, adsorption, xerogel, composite, silica, iron oxide

## Abstract

Vanadium is considered a strategic metal with wide applications in various industries due to its unique chemical and physical properties. On the basis of these considerations, the recovery of vanadium (V) is mandatory because of the lack of raw materials. Various methods are used to recover vanadium (V) from used aqueous solutions. This study develops a clean and effective process for the recovery of vanadium (V) by using the adsorption method. At the same time, this study synthesizes a material starting from silica matrices and iron oxides, which is used as an adsorbent material. To show the phase composition, the obtained material is characterized by X-ray diffraction showing that the material is present in the amorphous phase, with a crystal size of 20 nm. However, the morphological texture of the material is determined by the N_2_ adsorption–desorption method, proving that the adsorbent material has a high surface area of 305 m^2^/g with a total pore volume of 1.55 cm^3^/g. To determine the efficiency of the SiO_2_Fe_x_O_y_ material for the recovery of vanadium through the adsorption process, the role of specific parameters, such as the L-to-V ratio, pH, contact time, temperature, and initial vanadium concentration, must be evaluated. The adsorption process mechanism was established through kinetic, thermodynamic, and equilibrium studies. In our case, the process is physical, endothermic, spontaneous, and takes place at the interface of SiO_2_Fe_x_O_y_ with V_2_O_5_. Following equilibrium studies, the maximum adsorption capacity of the SiO_2_Fe_x_O_y_ material was 58.8 mg (V)/g of material.

## 1. Introduction

Vanadium is used to produce steel with rust-resistant elements and as a carbide stabilizer in the manufacture of steel, especially for nuclear applications. About 80% of the vanadium produced is used as an additive for stainless steel or ferranadium. For titanium steel plating, vanadium foil was used as a bonding agent. Vanadium pentoxide is used as a catalyst, as a mordant for dyeing and printing fabrics, in the manufacture of aniline black, and in the ceramics industry. Vanadium-gallium tape is used to produce superconducting magnets [1].

Vanadium can be found in about 65 minerals, including vanadinite (Pb_5_(VO_4_)_3_Cl), carnotite (K_2_(UO_2_)_2_(VO_4_)_2_**·**3H_2_O), patronite (VOSO_4_), and roscoelite (K(V^3+^,Al,Mg)_2_AlSi_3_O_10_(OH)_2_). In addition, vanadium can be found in certain iron and phosphate ores and in some crude oils as organic complexes. Vanadium is found in small percentages in meteorites. Vanadium is an important component of catalysts for oxidation reactions.

Typical applications of vanadium (V) are as a catalyst for SO_2_ oxidation, for the oxidation of propane and propene to acrylic acid [2,3,4], for oxidative dehydrogenation of butane [5], or for oxidation of butane to maleic anhydride [6]. It is also used for the selective catalytic reduction of NO_x_ from ammonia [7]. BiVO_4_ can also be used as a catalyst for the electrochemical synthesis of H_2_O_2_ [8].

Different vanadium compounds were founded in relatively low amounts in living organisms, being distributed in the bones, liver, and kidney [9]. In this context, polyoxidovanadates present pharmacological action, being tested as anticancer, antiprotozoal, antidiabetic, antibacterial, and antiviral drugs [10,11,12]. Starting from such research becomes evident that the proper disposal and further recovery of the vanadium ions from expired drugs are required.

As a result of all of these applications, relatively large amounts of vanadium (V) are accumulated in various industrial wastes. Given the value of vanadium (V), from an economic point of view, recovery has become a necessity. Various processes are being developed for this purpose. Recovery options include hydrometallurgical operations, such as leaching, purification of the solution by solvent extraction, and precipitation, and pyrometallurgical operations, such as direct melting and frying [13]. Ionic exchange could be another option for recovery [14].

A material has good adsorbent properties if it presents a high adsorption capacity, the smallest pore diameter, the largest specific surface area, and the possibility of high regeneration. Among the usual materials used are activated carbon, silica gel, alumina, zeolites, polymer matrix, etc. [15,16,17].

The goal of this study is to synthesize new materials that can have tuned properties based on silica and iron oxide. We obtained a SiO_2_Fe_x_O_y_ xerogel, which we further used to recover the vanadium from waste water by adsorption, indicating good results. This adsorbent material was chosen because similar material was used for recovery of different metallic ions, presenting an interesting behaviour. Another reason is represented by the presence of vanadium ions in red blood cells [9,11,18], which allows us to think that presence of iron can improve the adsorptive properties of this material.

## 2. Materials and Methods

### 2.1. Material Synthesis

Following previous work [19], SiO_2_Fe_x_O_y_ xerogel is synthesized using the sol-gel method, using tetraethyl-orthosilicate-TEOS, Si (OC_2_H_5_)_4_, (SigmaAldrich) (7 mL) as silica precursors and iron (II) acetylacetonate, C_10_H_14_FeO_4_, Fe(acac)_2_ (SigmaAldrich, Burlington, MA, USA) (0.54 g) as iron oxide precursors. The TEOS is dissolved in 37.2 mL of ethanol (Chimopar, SC CHIMOPAR TRADING SRL, Bucharest, Romania) and stirred for 10 min at 500 rpm using a magnetic stirrer (DLAB MS-HS); then, 13.8 mL of deionised water is added and stirred for 30 more minutes. Iron (II) acetylacetonate is also added, stirring mechanically for 3 h and maintaining a temperature of 55 °C in the solution. Gels are formed when NH_3_ (Chimopar, SC CHIMOPAR TRADING SRL) (0.46 g) is used as catalyst, with the pH becoming 10. Obtained material is left for 24 h to age at room temperature and then dried in an oven (POL-EKO SLW53, Poland) at 100 °C for 24 h.

**Figure 1:**
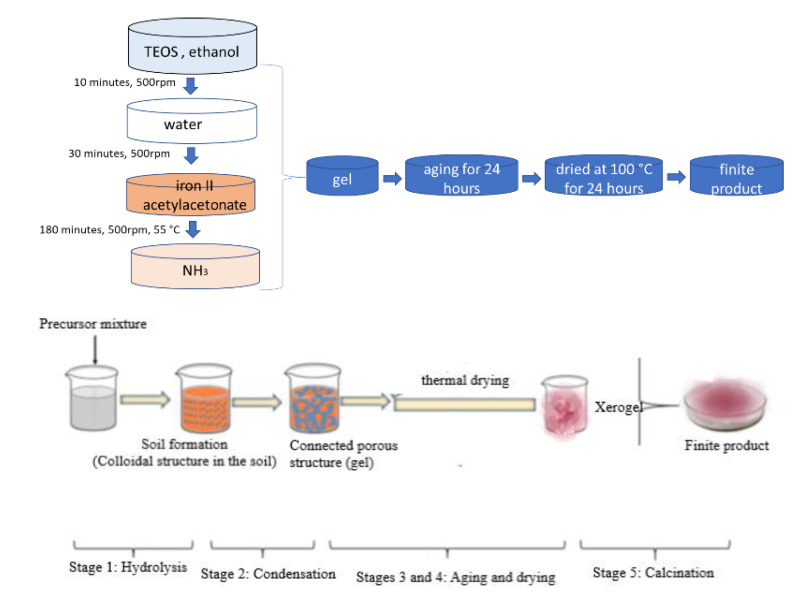
SiO_2_Fe_x_O_y_ xerogel synthesis scheme.

A schematic presentation of the synthesis is shown in Figure 1.

### 2.2. Material Characterization

Knowledge regarding material crystallinity degree, but also about the existence of several phases in the material, X-ray diffraction (XRD) analysis was performed using the Ultima IV instrument (RIGAKU) operating with Co-Kα radiation. N_2_ adsorption–desorption isotherm is obtained using the Nova 1200e Quantachrome apparatus. Furthermore, the newly prepared material was characterized by scanning electron microscopy (SEM) coupled with energy dispersive X-ray spectroscopy (EDX, Cambridge, MA, USA), employing the FEI Quanta FEG 250 scanning electron microscope.

### 2.3. Studies on the Recovery of Vanadium (V) Ions by Adsorption onto SiO_2_Fe_x_O_y_ Material

#### 2.3.1. Effect of the S:L Ratio

To establish the S:L ratio at which the best recovery efficiency of vanadium (V) takes place, the amount of adsorbent material, SiO_2_Fe_x_O_y_ (0.05; 0.1; 0.2; 0.3; 0.4; and 0.5), was varied, maintaining the constant volume of solution (25 mL) of ammonium metavanadate, NH_4_VO_3_ (Merck, Darmstadt, Germany), containing 50 mg Vanadium (V)/L. Adsorption was accomplished in a Julabo SW23 shaker with thermostat and stirring, where the samples are kept in contact for 60 min at 298 K and 200 rpm.

Vanadium (V) analysis was performed by UV-VIS spectroscopy using the Varian Carry 50 spectrometer. Then, 2.5 mL of sample solution was added to 1 mL of H_2_SO_4_ 6 N (CHEMICAL Company), 1 mL of H_3_PO_4_ 6 N (Sigma Aldrich) and 0.5 mL of Na_2_WO_4_ solution (Merck) (8.25 g of Na_2_WO_4_ in 50 mL of water). The spectra were recorded at a wavelength of 400 nm.

#### 2.3.2. pH Effect

To determine the pH influence on the vanadium adsorption process on the adsorbent material, 0.1 g of material, SiO_2_Fe_x_O_y_, was kept in contact for 60 min at a temperature of 298 K with 25 mL of solution at initial concentration, C_0_ = 50 mg V (V)/L. Solutions’ pH values were adjusted using solutions of HNO_3_ (63%, Carl Roth) and NaOH (Sigma Aldrich) solutions with concentrations in the range of 0.1–1 N.

#### 2.3.3. Contact Time and Temperature Effect

To study the influence of temperature and contact time on the adsorption capacity of the adsorbent material, 0.1 g of material was accurately weighed and over 25 mL of vanadium solution of initial concentration C0 = 50 mg/L were added. Using a water bath with thermostat and stirring, the xerogel sample was first stirred at 200 rpm for 15, 30, 60, and 120 min at three different temperatures, namely 298 K, 308 K, and 318 K.

#### 2.3.4. Kinetic Studies

The kinetic equations used to investigate kinetics of the vanadium adsorption process are presented in Table 1.

#### 2.3.5. Activation Energy, E_a_

The activation energy E_a_ was calculated using the Arrhenius equation and the speed constant from the pseudo- second order kinetic model k_2_ is represented graphically by ln k_2_ = f (1/T).

$$\ln k_{2}=\ln A-\frac{E_{A}}{R\;T}$$

where: k_2_—Speed constant, g/min∙mg

A—Arrhenius constant, g∙min/mg

E_a_—Activation energy, kJ/mol

T—Absolute temperature, K

R—The ideal gas constant, 8.314 J/mol∙K.

The activation energy, Ea, will give us the information needed to describe the nature of the adsorption process.

#### 2.3.6. Thermodynamic Studies

The temperature range of 298–318 K was selected to evaluate the thermodynamic studies by calculating the free energy using the Gibbs-Helmholtz equation [15]:

$$\Delta G^{0}=\Delta H^{0}-T\;\Delta S^{0}$$

where: ΔG^0^—standard Gibbs free energy variation, kJ/mol

ΔH^0^—standard enthalpy variation, kJ/mol

ΔS^0^—standard entropy variation, J/mol∙K

T—absolute temperature, K.

Using the van’t Hoff equation obtained from the linear representation of ln K_d_ = f (1/T), the standard variation of the entropy ΔS^0^ and the standard variation of the enthalpy ΔH^0^ can be calculated.

$$\ln K_{2}=\frac{\Delta S^{0}}{R}-\frac{\Delta H^{0}}{R\;T}$$

where: K_d_—equilibrium constant

ΔS^0^—standard entropy variation, J/mol∙K

ΔH^0^—standard enthalpy variation, kJ/mol

R—the ideal gas constant, 8.314 J/mol∙K.

The equilibrium constant is the ratio between the adsorption capacity at equilibrium q_e_ and the equilibrium concentration C_e_.

$$K_{d}=\frac{q_{e}}{C_{e}}$$

The plot ln K_d_ = f (1/T) will be represented.

#### 2.3.7. Initial Concentration Effect

To determine the effect of the initial Vanadium (V) concentration on the adsorption capacity of the SiO_2_Fe_x_O_y_ material, V (V) solutions of different concentrations, namely 25, 50, 75, 100, 120, 160, 200, 300, 400, 500, and 600 mg/L were prepared. These were obtained by an appropriate dilution from a stock solution of ammonium metavanadate, NH_4_VO_3_, 1000 mg/L. Adsorption was performed at the ratio S:L = 0.1 g:25 mL, pH = 2, contact time 60 min and at a temperature of 298 K.

#### 2.3.8. Equilibrium Studies

To establish the adsorption mechanism, three isotherms were used, namely Langmuir, Freundlich, and Sips (Table 2).

#### 2.3.9. Desorption Studies

The reprocessing of absorbents is an important economic factor. At the same time, obtaining a solution with concentrated vanadium (V) is essential for further applications. Therefore, adsorption–desorption studies have been performed to investigate the potential for reuse of SiO_2_Fe_x_O_y_ material. Initially, adsorption studies were performed at equilibrium, when 25 mL of vanadium solution (having initial concentration of 400 mg/L) were mixed with 1 g of adsorbent material at pH = 2, contact time 90 min and 298 K. Desorption experiments were performed using 50 mL of NaNO_3_ (Carl Roth) 5 M, and also NaCl (Merck, Germany) 5 M solutions, which were mixed with exhausted adsorbent material for 120 min. After time has elapsed, the desorbed vanadium content has been analysed.

## 3. Resultants and Discussion

### 3.1. Characterization of the Synthesized Material

#### 3.1.1. X-ray Diffraction (XRD)

**Figure 2:**
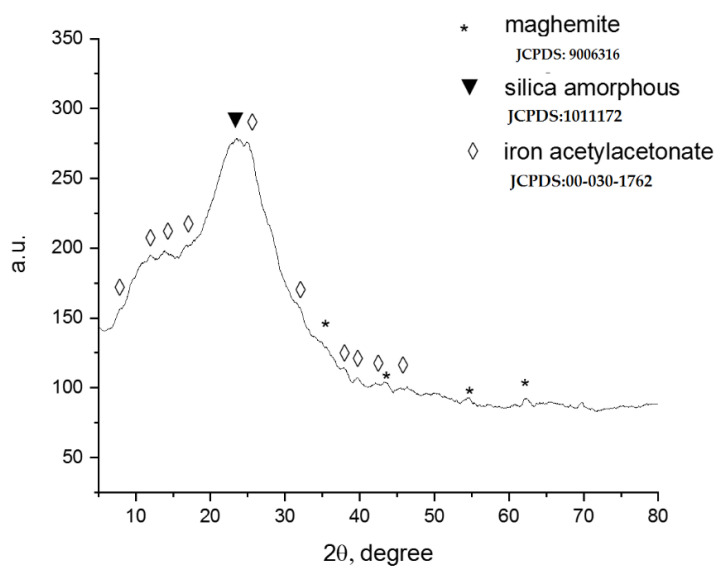
X-ray diffraction.

Figure 2 shows the spectrum obtained for SiO_2_Fe_x_O_y_ material.

The following databases were taken into account for the identification of the main phases in the material: JCPDS:9006316 specifics for the cubic phase Fd3m of maghemite, JCPDS:00-030-1762 specific for the orthorhombic phase for acetyl iron acetone II, and JCPDS:1011172 for amorphous silica.

The XRD diffractogram of the synthesized material shown in Figure 2 generally indicates an amorphous aspect of the sample. The diffraction lines specific to SiO_2_ observed at 20–30 2θ overlap with those specific to Fe(acac)_2_. Detailed analysis of the recorded spectrum also shows the appearance of diffraction lines at 35, 43, 57, 63 2θ, specific to the formation of γ-Fe_2_O_3_ but in a very small amount. According to Scherrer’s calculation, it was found that the γ-Fe_2_O_3_ particles were an average size of 20 nm.

#### 3.1.2. N_2_ Sorption Analysis

The determination of porosity is based on the measurement of the amount of gas (usually N_2_) adsorbed or desorbed on the surface of solids, porous or nonporous. The adsorbent material is maintained at a temperature below the critical temperature of the adsorbed, 77 K. During adsorption and desorption, the pressure changes until equilibrium is established. To analyse the sample, it is degassed under vacuum for 24 h at room temperature.

**Figure 3:**
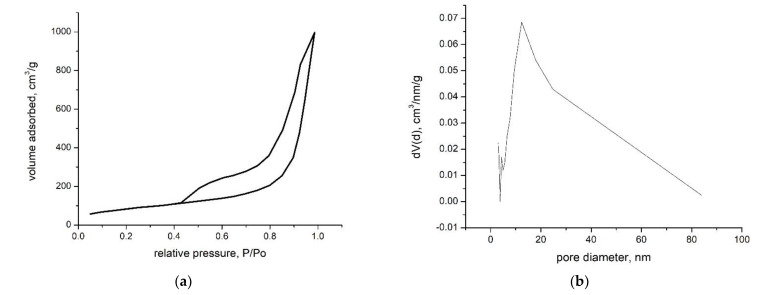
N_2_ adsorption-desorption isotherm for composite material (**a**) and pore size distribution (**b**).

In Figure 3a, the adsorption–desorption isotherm of the composite material, SiO_2_Fe_x_O_y_, is presented, and Figure 3b shows the pore size distribution obtained using BJH (Barrett, Joyner and Halenda) method from the desorption branch.

Analysing the data obtained and correlating with the IUPAC references [26], we notice that the analysed material has a specific behaviour to the type IVa isotherm. This type of isotherm has H3-type hysteresis. In this case, capillary condensation occurs faster, but not completely, and the material has pores in the macropore region.

By applying the BJH method for the desorption branch, the pore size distribution shown in Figure 3b can be obtained. The analysed sample indicates that the material has an average pores size of 12 nm, but, as we can see, macropores are also present (>50 nm).

From the adsorption branch, using the BET method (Brunauer, Emmet, Teller) in the range 0.05–0.3 P/Po, a specific surface of 305 m^2^/g is obtained. What is interesting is that the SiO_2_Fe_x_O_y_ material, even if it indicates a relatively small specific surface area, has a very large total pore volume, namely 1.55 cm^3^/g. The data from the literature show that the pore volume obtained is high, unlike other similar studies [27,28], which may lead us to expect good results in terms of vanadium recovery by adsorption on the synthesized material.

#### 3.1.3. Scanning Electron Microscopy (SEM) Analysis

**Figure 4:**
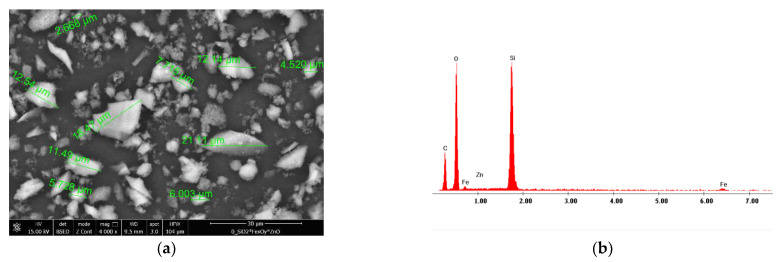
SEM picture recorded for prepared adsorbent material (**a**) and EDX spectra obtained for prepared adsorbent material (**b**).

In order to get the adsorbent morphology, the SEM pictures and the EDX spectra were recorded, which prove the predicted composition of newly prepared material (pictures are presented in Figure 4).

From the image presented in Figure 4a, it can be observed that the prepared adsorbent materials have a low degree of crystallinity, with several crystals with dimensions between 2 and 20 μm. Moreover, from the EDX spectrum presented in Figure 4b, we can observe that the prepared material has the expected composition.

#### 3.1.4. Studies on Vanadium Ions Recovery by Adsorption on SiO_2_Fe_x_O_y_ Material

Information regarding the adsorption behaviour of vanadium ions on synthesized xerogels is essential for understanding the mobility of vanadium (V) in aqueous systems. This study investigates the influence of some parameters (S:L ratio, pH, contact time, temperature, and initial concentration of V (V)) on the adsorption capacity of SiO_2_Fe_x_O_y_ material, but also the mechanism of the V adsorption process (V).

#### 3.1.5. Adsorbent Dose Effect on the Adsorption of Vanadium

**Figure 5:**
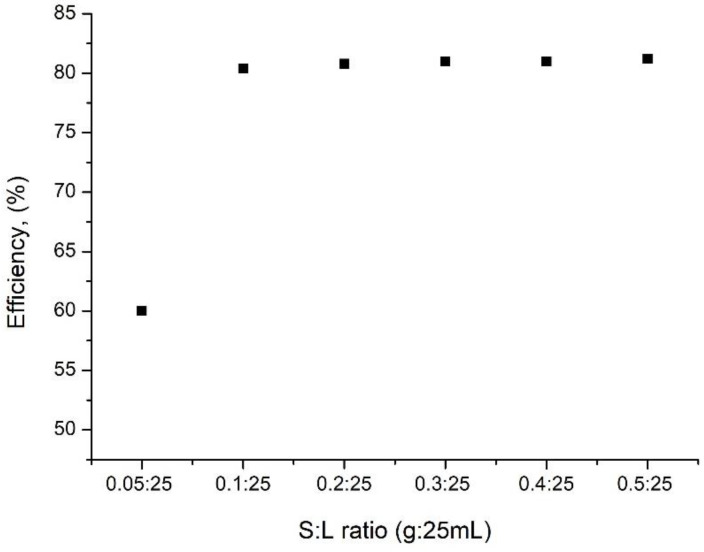
Effect of the dose of adsorbent on vanadium ions adsorption on the SiO_2_Fe_x_O_y_ material.

The effect of solid:liquid (S:L) ratio on the efficiency of the vanadium recovery process is shown in Figure 5.

It is observed that as the S:L ratio increases, the efficiency of the vanadium adsorption process onto the SiO_2_Fe_x_O_y_ material increases. Starting with the ratio S:L = 0.1 g of adsorbent material:25 mL solution, the efficiency increases, but not significantly (~80%), which determines that all subsequent studies will be carried out at this ratio.

#### 3.1.6. Effect of pH

**Figure 6:**
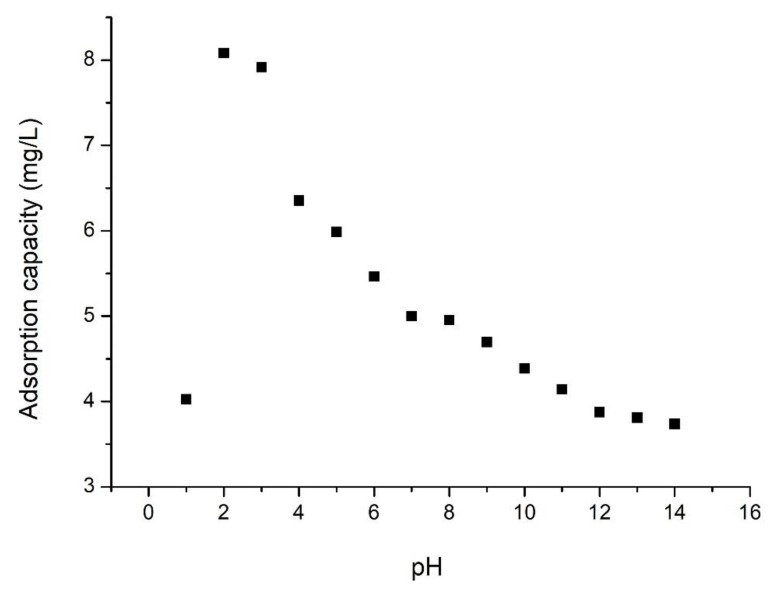
Effect of pH on the adsorption mount of vanadium (V).

Vanadium solution pH can affect the adsorption capacity of the prepared adsorptive material, being also studied. Experimental data obtained are presented in Figure 6.

From data presented in Figure 6, we can conclude that the highest adsorption capacity was obtained when the adsorption process was conducted at pH = 2 (~8 mg/g) [29].

**Figure 7:**
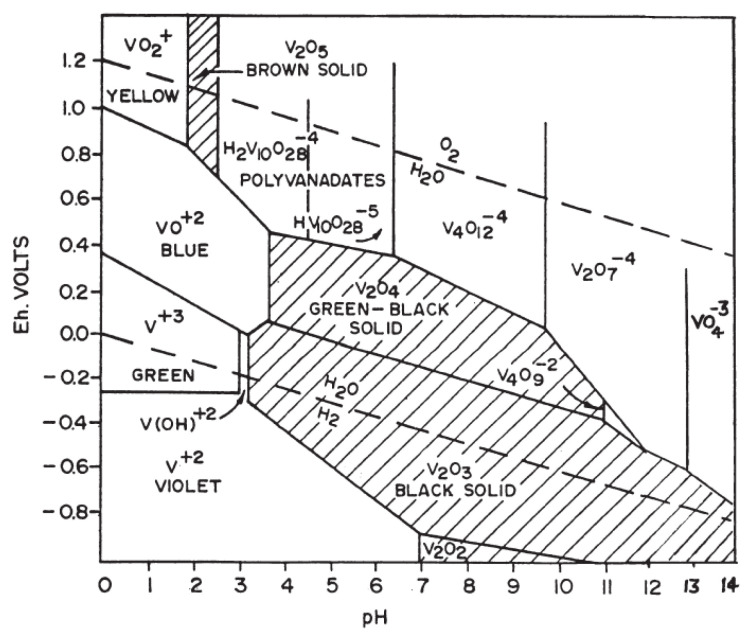
Relationship between the state of vanadium in aqueous solution and vanadium concentration and pH [30].

Figure 7 shows the Eh–pH diagram presenting the predominant species as a function of pH and redox potential.

Since the pH was set at pH = 2, which is acidic, and the colour of the solutions was yellow, according to Figure 7, the predominant species is ${\mathrm{VO}}_{2}^{+}$ (E° = 1.0 V) [30,31].

#### 3.1.7. Effect of Contact Time and Temperature

**Figure 8:**
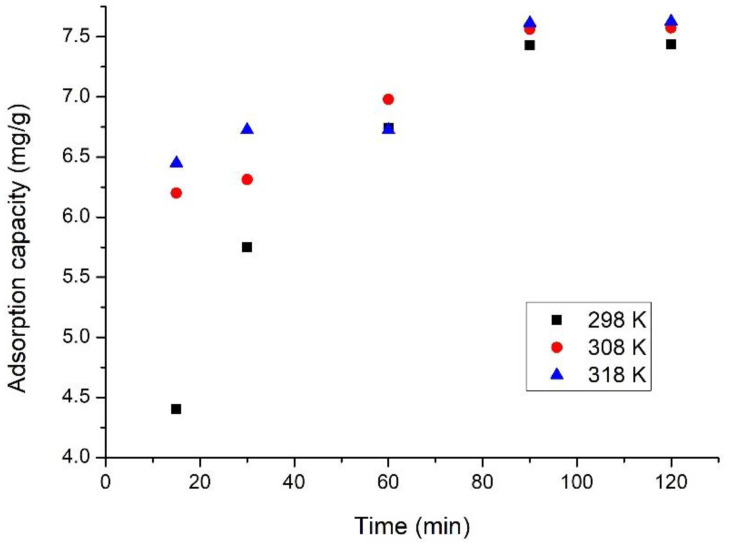
Effect of contact time and temperature on the V(V) recovery.

The effects of contact time and temperature are shown in Figure 8.

Data presented in Figure 8 indicate that with the increase of the contact time, the adsorption capacity of SiO_2_Fe_x_O_y_ material increases, up to 90 min. After a period of more than 90 min, the adsorption capacity of the material remains approximately constant (~7.5 mg V (V)/g of adsorbent material), leading us to the conclusion that a contact time of more than 90 min is not required. This behaviour of the adsorbent material can be explained only by considering that the adsorption process is mainly controlled by the inner surface diffusion of vanadium ions (V). Adsorption of vanadium ions from aqueous solution can be considered a long process, until the adsorption equilibrium is reached due to the adsorption of vanadium ions onto the porous material, controlled mainly by an inner surface diffusion process, being much slower than the bulk diffusion [32].

It is also observed that temperature could positively influence the adsorption capacity of the SiO_2_Fe_x_O_y_ material. Thus, the higher the temperature, the higher the adsorption capacity, such increase being not significant so long as the adsorptive proves being conducted at a temperature of 298 K.

#### 3.1.8. Kinetic Studies

To investigate the kinetics of the vanadium adsorption process on SiO_2_Fe_x_O_y_, obtained experimental data were modelled using pseudo-first order and pseudo-second order equations.

**Figure 9:**
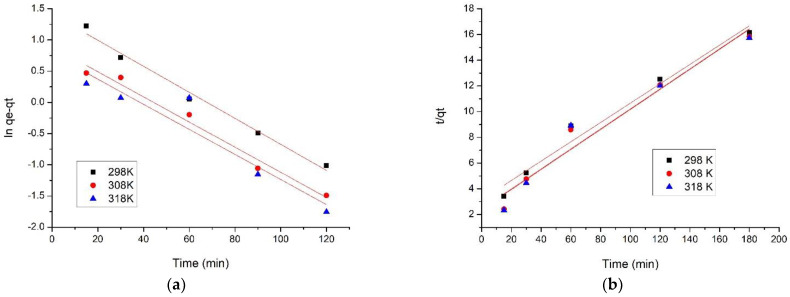
Pseudo-first order (**a**) and pseudo-second order isotherms (**b**).

The kinetics of the studied adsorption process on SiO_2_Fe_x_O_y_ adsorbent material were studied at three different temperatures. Thus, in Figure 9a,b, pseudo-first order and pseudo-second order isotherms are shown.

The adsorption process of V (V) on the studied material proceeds in three stages: (i) a rapid initial stage (the first 40 min), followed by (ii) a slower adsorption stage, and (iii) after 90 min of adsorption to reach balance. The rapid adsorption step can be attributed to easy activation and surface accessibility of the material, but since the sites are covered with V (V) ions, the adsorption rate decreases [33].

Speed constants values, calculated adsorption capacity values, as well as the values of the regression coefficient, R_2_, are presented in Table 3.

Analysing data presented in Table 3 can observe that obtained experimental data are well-modelled by the pseudo-second order kinetic model, supported the regression coefficient value close to 1 *R*^2^~1 (0.9999–0.9958). By modelling experimental data with the pseudo- first order kinetic model, *R*^2^ values range between 0.9884 and 0.9204. Additionally, data obtained from modelling experimental data with the pseudo-second-order model proved that q_e,calc_ has values close to q_e,exp_. An insignificant influence of temperature on the values of the parameters k_2_, q_e,calc_, can be observed, so it will be not necessary to work at temperatures higher than 298 K.

#### 3.1.9. Intraparticle Diffusion

The Weber–Morris intraparticle diffusion model was used to further investigate the mechanism of vanadium diffusion on the adsorbent and the controlling steps that affect the adsorption kinetics.

**Figure 10:**
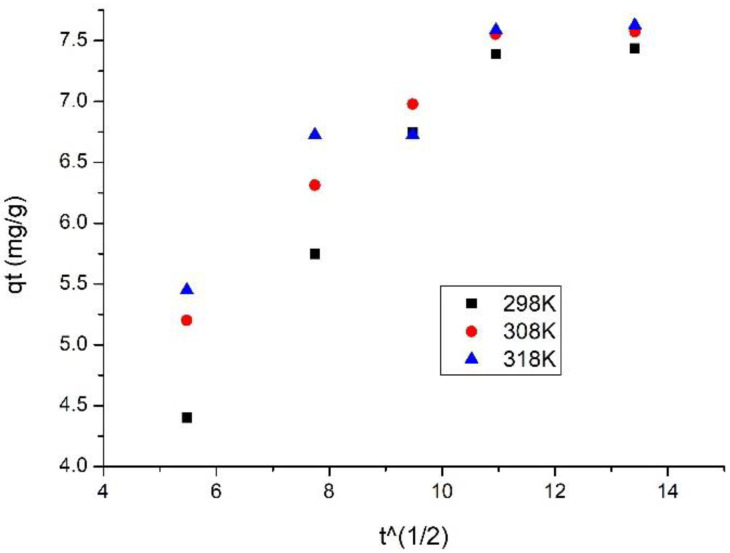
Intraparticle diffusion models for three different temperatures.

Figure 10 shows the Weber–Morris specific intraparticle diffusion model at 3 temperatures.

Analysing the data presented in Figure 10, we can observe that the lines obtained from the representation of dependence q_t_ versus t^1/2^ do not pass through origin, signifying that the vanadium adsorption mechanism is a multi-stage one. Based on that, we can conclude that adsorption kinetics is influenced by intraparticle diffusion and by film diffusion. After data modelling, we obtained the values for K_diff_ and C parameters. Data are presented in Table 4.

Taking into account data presented in Table 4, we can observe that, by temperature increase, value of K_diff_ increases. It is also observed that, for the first stage, diffusion constants are higher than the diffusion constant specific for stage 2, allowing us to conclude that the speed determinant is stage 1, stage 2 being much slower [34].

Adsorption of vanadium ions from the aqueous phase on the SiO_2_Fe_x_O_y_ adsorbent material can be considered to be a three-stage process: (1) transfer of the sorbent from the aqueous phase to the solid surface, (2) intraparticle diffusion into the pores of the adsorbent, and (3) adsorption at an internal site. Step (3) is assumed to be fast, whereas in terms of (1) and (2) there are steps that can be neglected in any kinetic analysis.

Considering the kinetics of the adsorption process, we can assume that the mass transfer resistance occurs in steps (1) and (2), and can be controlled by acting either individually or in combination. In this study, two kinetic models were presented, namely external mass transfer diffusion and intraparticle mass-transfer diffusion models, to describe the vanadium adsorption mechanism on SiO_2_Fe_x_O_y_ [32,35].

#### 3.1.10. Activation Energy

**Figure 11:**
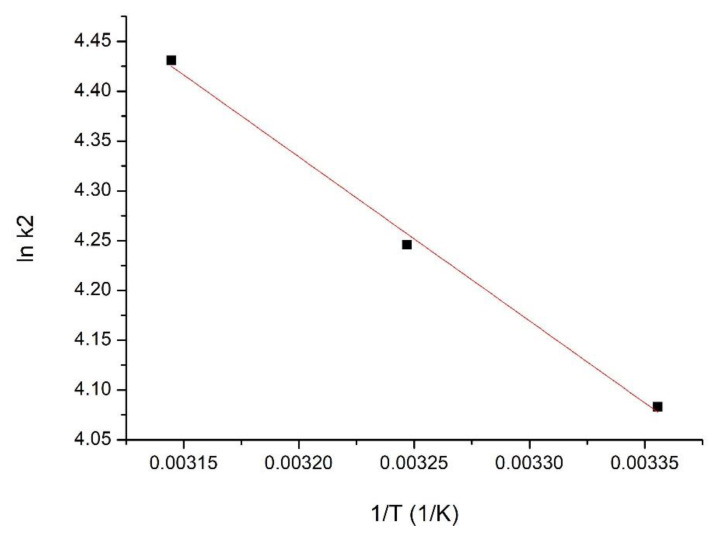
Ln k_2_ vs. 1/T plot for SiO_2_Fe_x_O_y_ material.

The adsorption activation energy V (V) on the SiO_2_Fe_x_O_y_ material was evaluated based on the associated equation of the graphical representation ln k_2_ = f (1/T) (Figure 11).

It is observed that the activation energy value (13.7 kJ/mol) is <40 kJ/mol; from this value, we can conclude that the studied adsorption process is a physical one [31].

#### 3.1.11. Thermodynamic Studies

**Figure 12:**
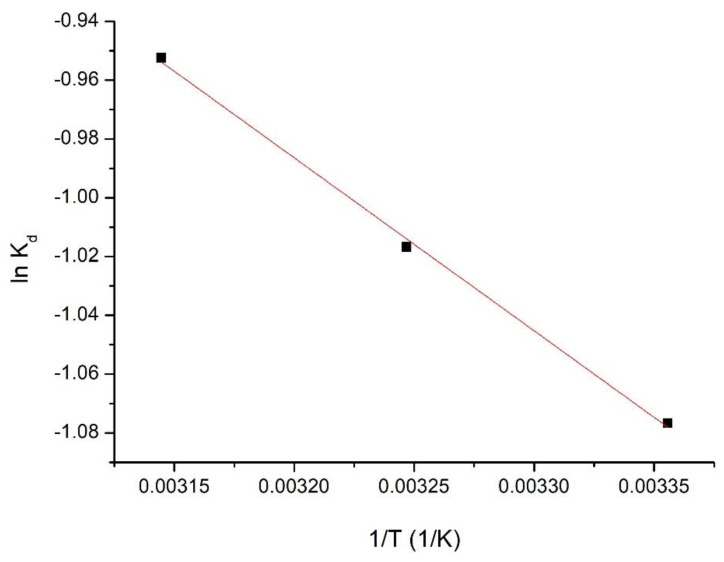
Thermodynamic studies for determination of ln K_d_ vs. 1/T plot for SiO_2_Fe_x_O_y_ sample.

Thermodynamic studies were performed in the temperature domain between 298 to 318 K. Figure 12 shows the dependence ln K_d_ = f (1/T).

Table 5 presents the calculated thermodynamic parameters at all three temperatures.

Upon evaluation of the resulting data, the positive value of ΔH^0^ means that the adsorption process is endothermic.

It is also observed that ΔG^0^ has negative values and increases in absolute value with temperature increase, indicating a spontaneous adsorption process, which is influenced by temperature.

The fact that the value of ΔS^0^ is positive indicates that the adsorption process is favoured, taking place at the interface of the SiO_2_Fe_x_O_y_ material/solution with V (V).

#### 3.1.12. Initial Concentration Effect

**Figure 13:**
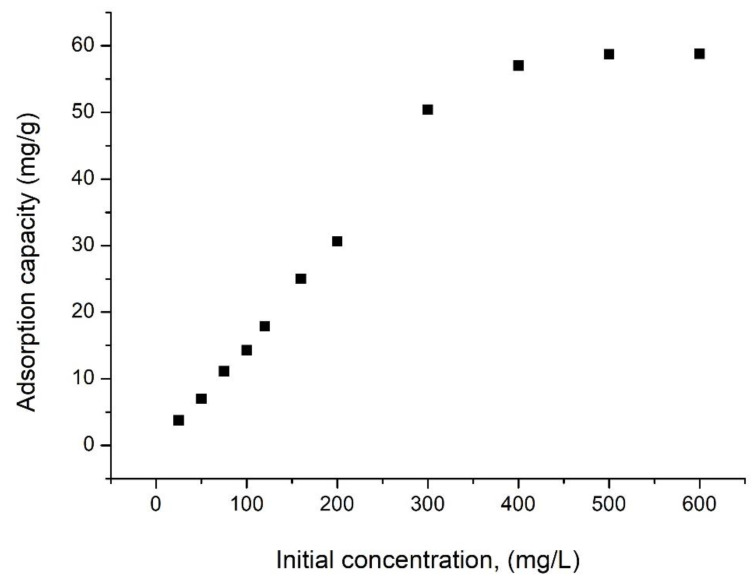
Effect of initial concentration on the adsorption capacity for SiO_2_Fe_x_O_y_ sample.

The effect of the initial concentration of V_2_O_5_ on the adsorption capacity of the material is shown in Figure 13.

From data presented in Figure 13, we can observe that as the initial concentration of V (V) increases, the adsorption capacity increases. At concentrations greater than 400 mg V (V)/L, the adsorption capacity remains constant (q~58.8 mg V (V)/g), being considered the maximum adsorption capacity of the material.

#### 3.1.13. Equilibrium Studies

**Figure 14:**
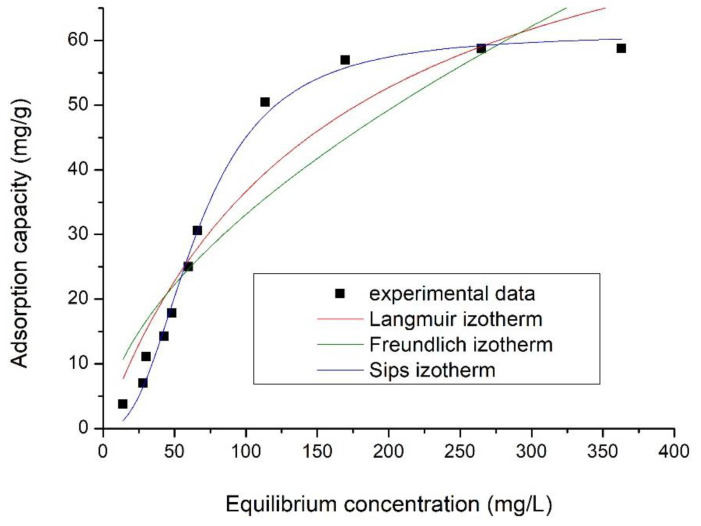
Equilibrium isotherms for SiO_2_Fe_x_O_y_ sample.

Figure 14 presents the adsorption isotherms obtained by the graphical representation of q_e_ = f (C_e_).

Specific parameters associated with each adsorption isotherm used to model obtained experimental data were calculated from slopes of the straight line, and respectively, using the ordinate from the origin (Table 6).

Data presented in Figure 14 present the correlation between vanadium equilibrium concentration (C_e_) and adsorption capacity, proving that as the equilibrium concentration increases, the adsorption capacity increases until equilibrium is reached, establishing the maximum adsorption capacity obtained experimentally, q_e,exp_.

Analysing data presented in Table 6, we can observe that the studied adsorption process is well-described by the Sips model, as the regression coefficient obtained in this case, *R*^2^, is closest to 1 (*R*^2^ = 0.9914).

The studied adsorption process is homogeneous one. In this case, adsorption occurs through the interaction of a solute molecule with an active centre on the surface of the sorbent. Adsorption occurs on the surface of the sorbent, resulting in a monolayer, the solute molecules being retained only on the free surface of the adsorbent [32].

A comparison of the adsorption capacity of prepared adsorbent material with the adsorption capacity obtained for some other materials is presented in Table 7. From presented data, we can observe that the prepared SiO_2_Fe_x_O_y_ material has good adsorption capacity.

#### 3.1.14. Desorption Studies

Adsorbent material has been regenerated using two different solutions (NaNO_3_ and NaCl) [31] with a concentration of 5 M. When NaNO_3_ 5 M was used for regeneration, during the first adsorption/desorption cycle, 75% of adsorbed vanadium ions were desorbed. Further, in the second adsorption/desorption cycle, 67% of adsorbed vanadium was desorbed. In the last adsorption desorption cycle (fourth one), only 38% of adsorbed vanadium was desorbed.

When NaCl was used for the regeneration of the adsorbent material, it was observed that during the first adsorption/desorption cycle, only 57% of adsorbed vanadium had been desorbed. In the third cycle, only 36% of adsorbed vanadium was desorbed, so we can consider that in this case the adsorbent material was exhausted. Based on these observations, we can conclude that the newly prepared adsorbent material can be regenerated using NaNO_3_ and reused for four times in the vanadium recovery process.

## 4. Conclusions

The following conclusions can be drawn from the studies presented. In this study, a new material with tuned properties was synthesized, obtaining a composite xerogel based on silica matrices and iron oxides, SiO_2_Fe_x_O_y_, which proved to be effective for vanadium ions recovery from aqueous solutions by adsorption. Prepared adsorbent material was characterized by X-ray diffraction and N_2_ sorption analysis.

Vanadium recovery process takes place under the following conditions: S ratio:L = 0.1 g:25 mL, pH = 2, contact time 90 min, and 298 K; studies being performed in batch. Based on obtained results, kinetic, thermodynamic, and equilibrium studies were performed. Kinetically, the pseudo-second order isotherm model is the one that best describes the process.

In order to prove if the determinant step of speed is the film diffusion or the intraparticle diffusion, kinetic experimental data were modelled using the Weber–Morris model. The porous structure of SiO_2_Fe_x_O_y_ material allows the adsorption sites to be placed onto the surfaces located inside the adsorbent channels, which indicates that vanadate can be adsorbed on the surface of SiO_2_Fe_x_O_y_ (stage I), and further reaches equilibrium (stage 2), meaning that intraparticle diffusion is not a speed limiting step during adsorption. Adsorption is performed in films. The activation energy, Ea, was also determined by the fact that the adsorption process is of a physical nature.

Based on thermodynamic studies, we can conclude that the adsorption process is an endothermic, and spontaneous, being influenced by temperature, and that the adsorption process takes place at the interface of the SiO_2_Fe_x_O_y_ material/V (V) solution.

Taking in account performed equilibrium studies, it was observed that the Sips model best describes the adsorption process, establishing that the maximum adsorption capacity of the SiO_2_Fe_x_O_y_ material was 58.8 mg of V ions/g of adsorbent material.

Desorption studies confirm that the adsorption process is controlled by pH, so that if the process proceeds at pH = 2, the material can be recovered and reused with 75% efficiency.

Based on our research, we can conclude that prepared adsorbent material can be used for vanadium recovery from aqueous solutions, with the adsorptive process being endothermic and spontaneous.

## Figures and Tables

**Table 1:** Kinetic equations.

Model	Equation	Reference
Pseudo-first order model (Lagergren model)	$\ln\left(q_{e}-{\;q}_{t}\right)=\ln q_{e}-{\;k}_{1}\;t$ where: q_e_—equilibrium adsorption capacity, mg/g q_t_—adsorption capacity at t time, mg/g k_1_—speed constant for pseudo-first order equation, 1/min t—contact time, min.	[20]
Pseudo-second order model (Ho și McKay model)	$\frac{t}{q_{t}}=\frac{1}{k_{2}{\;q}_{e}^{2}}+\frac{t}{q_{e}}$ where: q_e_—equilibrium adsorption capacity, mg/g q_t_—adsorption capacity at t time, mg/g k_2_—speed constant for pseudo-second order equation, g/mg∙min t—contact time, min.	[21]
Intraparticle diffusion (Weber and Morris model)	q_t_ = k_diff_ · t^1/2^ + C where: q_t_—adsorption capacity at t time, mg/g k_diff_—speed constant for intraparticle diffusion, mg/g·min^1/2^ C—constant correlated with the thickness of the liquid film surrounding the adsorbent particles.	[22]

**Table 2:** Equilibrium isotherms.

Isotherms Model	Equation	Reference
Nonlinear Langmuir isotherm	$q_{e}=\frac{q_{L}K_{L}C_{e}}{1+K_{L}C_{e}}$ where: q_L_—Langmuir maximum adsorption capacity, mg/g K_L_—Langmuir constant C_e_—equilibrium concentration, mg/L Q_e_—equilibrium adsorption capacity, mg/g	[23]
Linear Langmuir isotherm	$\frac{C_{e}}{q_{e}}=\frac{1}{q_{L}K_{L}}+\frac{C_{e}}{q_{L}}$ where: q_L_—Langmuir maximum adsorption capacity, mg/g K_L_—Langmuir constant C_e_—equilibrium concentration, mg/L Q_e_—equilibrium adsorption capacity, mg/g	[23]
Nonlinear Freundlich isotherm	$q_{e}=K_{F}C_{e}^{1/n_{F}}$ where: K_F_ and n_F_—characteristic constants that may be related to the relative adsorption capacity of the adsorbent and the adsorption intensity C_e_—equilibrium concentration, mg/L	[24]
Linear Freundlich isotherm	${\log\;q}_{e}={\;\mathrm{logK}}_{F}+1/n_{F}{\mathrm{logC}}_{e}$ where: K_F_ and n_F_—characteristic constants that may be related to the relative adsorption capacity of the adsorbent and the adsorption intensity C_e_—equilibrium concentration, mg/L	[24]
Nonlinear Sips isotherm	$q_{e}=\frac{q_{S}K_{S}C_{e}^{1/n_{S}}}{1+K_{s}C_{e}^{1/n_{S}}}$ where: K_S_—constant related to the adsorption capacity of the adsorbent, n_S_—heterogeneity factor C_e_—equilibrium concentration, mg/L	[25]

**Table 3:** Kinetic parameters.

**Pseudo-First Order**				
**Temperature (K)**	**q** **_e,exp_** **(mg/g)**	**k** **_1_** **(min^−1^)**	**q** **_e,calc_** **(mg/g)**	***R*** **^2^**
298	7.18	0.0108	4.10	0.9884
308	7.45	0.0185	6.54	0.9694
318	7.48	0.0201	7.18	0.9204
**Pseudo-Second Order**				
**Temperature (K)**	**q** **_e,exp_** **(mg/g)**	**k** **_2_** **(g/mg∙min)**	**q** **_e,calc_** **(mg/g)**	***R*** **^2^**
298	7.18	5.93	7.24	0.9999
308	7.45	6.98	7.40	0.9983
318	7.48	8.40	7.69	0.9958

**Table 4:** Specific parameters of intraparticle diffusion for SiO_2_Fe_x_O_y_.

Intraparticle Diffusion Model			
Temperature (K)	K_diff_ (mg/g·min^1/2^)	C	*R* ^2^
298	0.27	2.57	0.8539
308	0.31	3.80	0.8543
318	0.37	4.26	0.8169

**Table 5:** Thermodynamic parameters.

ΔH^0^, kJ/mol	ΔS^0^, J/mol∙K	ΔG^0^ kJ/mol			*R* ^2^
19.76	82.28	298 K	308 K	318 K	0.9985
		−4.75	−5.57	−6.40	

**Table 6:** Isotherm parameters.

**Langmuir Isotherm**			
q_m,exp_ (mg/g)	K_L_ (L/mg)	q_L_ (mg/g)	*R* ^2^
58.75	0.0064	93.95	0.9120
**Freundlich Isotherm**			
K_F_ (mg/g)	1/n_F_		*R* ^2^
2.35	0.57		0.8347
**Sips Isotherm**			
K_S_	q_S_ (mg/g)	1/n_S_	*R* ^2^
2.8·10^−5^	61.0	1.4	0.9914

**Table 7:** Adsorption performance comparison.

Materials	q, mg/g	References
montmorillonite	0.98	[33]
kaolinite	0.78	[33]
Aluminum-pillared Bentonite, Al-PILC	24.16	[32]
FeOOH	45.6	[31]
PGTFS-NH_3_^+^Cl^−^	45.86	[36]
Zr(IV)–SOW	56.75	[37]
ZnCl_2_ activated carbon	24.9	[38]
Metal hydroxide adsorbents: TiO_2_	25.06	[39]
SiO_2_Fe_x_O_y_	58.8	This study
